# Modeling Trap-Awareness and Related Phenomena in Capture-Recapture Studies

**DOI:** 10.1371/journal.pone.0032666

**Published:** 2012-03-02

**Authors:** Roger Pradel, Ana Sanz-Aguilar

**Affiliations:** Biostatistics and Population Biology Group, Centre d'Ecologie Fonctionnelle et Evolutive, Centre National de la Recherche Scientifique, Montpellier, France; University of Alberta, Canada

## Abstract

Trap-awareness and related phenomena whereby successive capture events are not independent is a feature of the majority of capture-recapture studies. This phenomenon was up to now difficult to incorporate in open population models and most authors have chosen to neglect it although this may have damaging consequences. Focusing on the situation where animals exhibit a trap response at the occasion immediately following one where they have been trapped but revert to their original naïve state if they are missed once, we show that trap-dependence is more naturally viewed as a state transition and is amenable to the current models of capture-recapture. This approach has the potential to accommodate lasting or progressively waning trap effects.

## Introduction

Live-trapping is a fundamental tool in the study of wildlife species and populations. When different trapping methods are used, empirical studies have found that different devices tend to catch different individuals [1]–[3]. While trappability with a particular device can sometimes be related to an identifiable feature (sex, age, weight [1], temperament [4]), this is not always possible. There is also evidence that knowledge of the trap fades with the passing of time [2]. The trap response issue is thus particularly acute when intervals between trapping occasions are short as is the case in closed population studies aiming at estimating population size. In these studies, it is generally considered that once an individual has been captured, its trappability changes for the rest of the study. Because trap response in this context is strong and because population size tends to be largely underestimated when the phenomenon is ignored, most work has been devoted to correcting for it in closed population models [5]–[7]. On the other hand, trap response in open population studies where occasions are generally separated by long intervals, typically a year, is much less considered. Yet, although the phenomenon is probably less intense, underestimation of survival is a true risk [8]. In this paper, we focus on short-time trap response in open populations, namely response affecting trappability solely at the occasion following one when the animal was trapped. This situation results in successive capture events being correlated and can be detected by appropriate tests—‘Test 2.CT’ for data from a single site [9] and ‘Test M.ITEC’ for multisite or multistate data [10]. However, the reciprocal is not true. With the above tests, trap dependence between successive occasions has been found when animals are captured in baited traps (trap-dependence *stricto sensu*) (e.g. [11]–[13]) but also in studies where individuals are not physically captured (trap-dependence *lato sensu*). Some situations where trap-dependence *lato sensu* occurs are: 1) When observers tend to visit some parts of the study area more often when marked individuals have been detected [14]–[15]; 2) When some patches of a heterogeneous habitat are more accessible so that individuals stationed there have higher resighting probabilities [16]–[17]; 3) When age, sex or social status are unknown, but determine individual movements or activity patterns so that the susceptibility to be recaptured or resighted varies [18]–[19]; 4) Or when non random temporary emigration occurs [8], often in relation to skipped reproduction [20]–[22]. For simplicity, we speak hereafter of ‘trap dependence’ to designate any correlation between capture events whatever its nature, as it is difficult to know for sure what type of trap dependence is at play in a particular study.

A survey of the literature shows that trap-dependence is a frequent phenomenon (Appendix S1). However, although the corresponding tests are largely available (program U-CARE, [23]), not all studies examine trap-dependence and it is not always clear whether this has been done in a particular study. Taking as a yardstick the papers citing Pradel (1993) where details of the way to detect and model trap-dependence in open populations were first expounded, the prevalence of trap dependence can be estimated at 71% (94/133) and touches several animal groups: birds, mammals, reptiles, amphibians, fish and insects (see Appendix S1). As for its nature, 32 papers put forward no interpretation, 26 evoke temporary emigration, 16 trap response, 8 individual heterogeneity, 7 the sampling protocol (biased sampling of known nests [14]–[15], unequal nest accessibility [3], [24]) and 5 some behavioral feature not directly related to the trap such as dominance. For some, in particular those evoking individual heterogeneity, the restriction of sighting dependence to one occasion may be too crude an approximation; specific models would be more appropriate [25]. Similarly, there exist specific models for temporary emigration [26]. Remarkably, only 76 of the 94 studies went on to incorporate trap-dependence at the data analysis stage. The method originally proposed to model trap-dependence [9] is indeed cumbersome and unnatural as a single individual has to be represented by several capture histories. In particular, it is difficult to combine with age-dependency. Another approach using individual covariates to code for capture at the preceding occasion [27]–[28] is probably more natural but still uneasy to put in practice. We propose here a new approach where trap-dependence is modeled as a change of state allowing it to be naturally incorporated in the current capture-recapture models [29].

## Methods

### Immediate Trap Effect seen as an animal state

Here, we describe the implementation of the basic Immediate Trap Effect on Capture model (ITEC, [9]) using trappability states. This model assumes that, when an animal is caught, it becomes aware of the trap and, depending on the case, will seek it or try to avoid it at the next occasion. However, if it is not caught, it reverts immediately to the ‘trap unaware’ state. This model is best described by examining the state of the animal at the end of each recapture session (denoted t^+^ when the precise timing need be specified) and how this state changes from one session to the next (alternatively, it is possible to consider the state at the beginning of each recapture session, but this approach would cause difficulties in the treatment of censored individuals, a situation frequently encountered). The individual is actually moving back and forth in a Markovian way between the state ‘trap aware’ (A) –its original state when it is first released after marking– and the state ‘trap unaware’ (U) which follows any occasion where it is not captured. At one point, the animal may also enter the state ‘dead’ (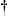), never to leave it again. To describe the capture histories under this model, we need three kinds of parameters: survival probabilities between capture sessions (φ), capture probabilities of trap aware individuals (p′), and capture probabilities of trap unaware individuals (p). Several kinds of dependency may be considered on these parameters (e.g., constancy, time or age dependency or individual characteristics, etc.) but the treatment of trap-dependence remains the same. Hence, for simplicity, we present the model as if parameters were constant.

The transition matrix, 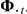 from the state at *t^+^* (in line) to the state at *t+1^+^* (in column) can be written as
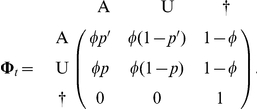
But it may be useful to separate the survival process (S), which takes place between times t^+^ and t+1^−^ (i.e. the instant just before occasion t+1) from the trap awareness process (P) assumed to take place between t+1^−^ and t+1^+^. Below, the time is specified as an index.
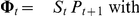


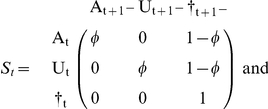


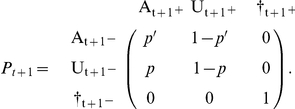
This model can be implemented as a multievent model [29] in program E-SURGE [30] or as a state-space model [31]–[32]. We detail here the first approach. Besides the transitions between states, the multievent formulation, which has a hidden Markov model structure, requires that probabilities of initial states be specified along with probabilities of the two events (‘encountered’, ‘not encountered’) conditional on the underlying state. However, initial state, assessed at the time of initial release, is necessarily ‘trap aware’ (A). As for the event probabilities, they are also trivial. If an animal is trap-aware at t^+^, that means that it has just been captured (conventional code ‘1’). If it is trap unaware or dead, it has not been captured during this session (conventional code ‘0’). This is summarized in the following matrix of event probabilities (E) with states in row and events in column.
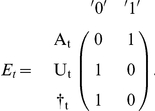
Using this approach, we were able to reproduce an analysis of survival of Cory's shearwaters (*Calonectris diomedea*) in presence of temporary emigration [20]. The new multievent approach proved strictly equivalent to the old approach where capture histories had to be split after each capture (Table 1). Table 1 also shows that ignoring trap-dependence would have led to an underestimation of survival. The practical implementation in program E-SURGE of model 5 of Table 2 in Sanz-Aguilar et al. (2011) is given in Appendix S2.

**Table 1:** An example of incorporating trap-dependence in capture-recapture models.

Model	(φ_1_,φ_2_, p_t_)	(φ_1_,φ_2_, p_t+m_)	(φ_1_,φ_2_, p_t+m_)
	no treatment	new approach	traditional approach
	of trap dependence	(trap-awareness states)	(split capture histories)
φ_1_	0.75 (0.69–0.80)	0.77 (0.70–0.82)	0.77 (0.70–0.82)
φ_2_	0.84 (0.80–0.87)	0.87 (0.82–0.90)	0.87 (0.82–0.90)

The current approach to modelling trap-dependence is compared to the traditional approach and to the model that ignores trap-dependence in a survival analysis of Cory's shearwaters (from [10]). Because there are transient individuals in this data set, two survival values are estimated: φ_1_, the apparent survival of newly-marked individuals, which is affected by the presence of transients, and φ_2_, the survival of previously marked individuals. Capture probability p is time-dependent-only in model (φ_1_,φ_2_, p_t_) and time- and trap-dependent in model (φ_1_,φ_2_, p_t+m_). In this last model, trap and time dependencies are additive. This model was fitted with the current approach, which considers trap-awareness states and with the traditional approach as in ([10] Model 5, Table 2), which involves the special preparation of the data detailed in [12]. The 95% confidence intervals are in parentheses.

For more complex situations where there are several types of observations, probabilities associated to each type of observation appear in the event matrix [29]. Appendix S2 contains such an example.

### Immediate Trap Effect with several sites or states

Most often, an analysis will involve state considerations, such as the breeding status or the geographical location. We treat here the multistate version of the ITEC model where, further to being ‘trap aware’ or ‘trap unaware’, individuals support another state classification. Without loss of generality, we assume that there are only two ‘other’ states. When combined with ‘aware’ and ‘unaware’, this leads to 4 (live) operational states: ‘aware’ and ‘unaware’ in state 1 (A1 and U1 respectively); ‘aware’ and ‘unaware’ in state 2 (A2 and U2 respectively). To which we add the state ‘dead’ (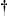). In what follows, we reserve the term ‘state’ for the states of ‘interest’. In addition to survival and capture probabilities as in the previous section, we consider now transition probabilities ø between the states of interest 1 and 2. In what follows, all parameters are explicitly shown as state-specific. Like in the single-site case, the model can be summarized through a transition matrix.
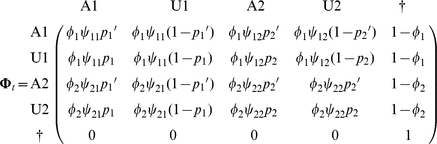
For instance, an individual which is in state 1 and is trap aware at t (operational state A1) may survive, remain in state 1, and not be caught at occasion t+1. In which case, it reverts to being trap unaware at t+1^+^. Its operational state at this moment changes to U1. The associated probability 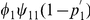 is found in row 1, column 2 of matrix 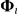. Now, it may be more illuminating to consider 3 steps: the survival process (S), which takes place between times t and t+1^−^, the state transition process (T), assumed to take place by the end of the interval at t+1^−^, and eventually the trap awareness process (P) assumed to take place between t+1^−^ and t+1^+^. Again, the time is specified through an index.
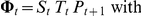


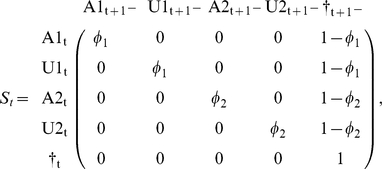


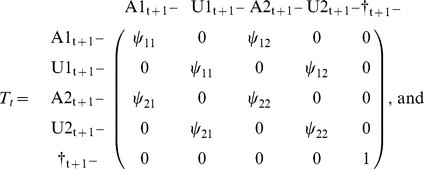


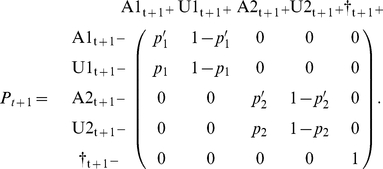
When an individual is initially released, it is trap aware but may be in state 1 or 2. There is thus a probability 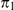 that it is in state 1 (operational state A1) and the complementary probability 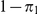 that it is in state 2 (operational state A2). This is summarized by the following vector of initial state probabilities.
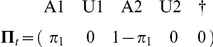
Like in the single state case, the specification of event probabilities is trivial. Like in the previous section, the code will necessarily be ‘0’ (not encountered) for trap-unaware and dead individuals. As for trap-aware individuals, we assume here that their state is recognized without error: ‘1’ for individuals in state 1, ‘2’ for individuals in state 2.
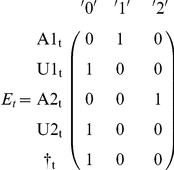
For a practical implementation of this model with program E-SURGE, see Appendix S2.

## Discussion

In the above models, unlike in traditional multistate capture-recapture models, capture probabilities appear among the transitions. This may be surprising to those used to the traditional models but is perfectly understandable when one realizes that, in presence of trap-dependence *stricto sensu*, the capture process does effect a change of state: after being captured, the animal knows of the trap and will adapt its behavior; the capture probability is thus a legitimate transition probability. In cases of trap-dependence *lato sensu* (overlap of survey area with territory, dominant individual with a conspicuous behavior, reproductive skipping, etc.), the capture event does not truly effect a change of state, but rather unveils a preexisting state (e.g. [20]). In these cases, dependence among sighting probabilities may well extend beyond one occasion, the extreme being intrinsic individual heterogeneity where the same individuals are always the highly catchable. For this last case, mixture models [27] are clearly more appropriate. One-step dependence and fixed heterogeneity represent actually two extremes of a gradient where the correlation lasts a more or less long time and may weaken progressively. With genuine trap response, this can be related to fading memory. When correlation is due to the overlap of the survey area with the individual territories, it may also be lost over time if territories and sampling protocol evolve progressively. The proposed approach could be extended to treat such cases by introducing appropriate holding times in the trap-aware state (semi-Markov process). At the moment, we recommend that in the absence of a clear understanding of the situation in a particular study where the tests for trap-dependence are significant, both immediate trap dependence and mixture models be tried. Temporary emigration models may accommodate intermediate situations even when transitions do not correspond to geographical movements.

## Supporting Information

Appendix S1 **Studies investigating trap-dependence.** Studies citing Pradel (1993) in which a trap-dependence effect has been found (research on ISI Web of Knowledge).
(DOCX)

Appendix S2 **Practical implementation of multievent trap-dependence models with program E-SURGE:** a medium-term monitoring program on Cory's Shearwater (*Calonectris diomedea*) as a case example.
(DOCX)
