# Correction: Modeling Trap-Awareness and Related Phenomena in Capture-Recapture Studies

**DOI:** 10.1371/annotation/e240f425-0375-4c32-b0a7-85fa586d0f40

**Published:** 2012-06-13

**Authors:** Roger Pradel, Ana Sanz-Aguilar

There was an error in the first paragraph of the Immediate Trap Effect with several sites or states section of Methods. The correct text is: In addition to survival and capture probabilities as in the previous section, we consider now transition probabilities ψ between the states of interest 1 and 2. 

