# Supplementary material for: Modeling Trap-Awareness and Related Phenomena in Capture-Recapture Studies
Source: PLoS One. 2012 Mar 2;7(3):e32666. doi: 10.1371/journal.pone.0032666 (PMC3292565; doi:10.1371/journal.pone.0032666)
Supplement: Appendix S1 — Studies investigating trap-dependence. Studies citing Pradel (1993) in which a trap-dependence effect has been found (research on ISI Web of Knowledge). (DOCX) [file pone.0032666.s001.docx]

**Appendix S1**

**Studies investigating trap-dependence**

***Studies citing Pradel (1993) in which a trap-dependence effect has been found (research on ISI Web of Knowledge).***

| Species group | Number of studies treating trap-dependence | Number of studies not treating trap-dependence |
| --- | --- | --- |
| Birds | 52 (83.87%) | 10 (16.13%) |
| Mammals | 18 (82.82%) | 4 (18.18%) |
| Reptiles | 2 (40%) | 3 (60%) |
| Amphibians | 2 (100%) | 0 |
| Fish | 1 (50%) | 1 (50%) |
| Insects | 1 (100%) | 0 |
| TOTAL | 76 (80.85 %) | 18 (19.14 %) |

Bird studies correcting for trap-dependence

Bachler E, Schaub M (2007) The effects of permanent local emigration and encounter technique on stopover duration estimates as revealed by telemetry and mark-recapture. Condor 109: 142-154.

Balanca G, Schaub M (2005) Post-breeding migration ecology of Reed Acrocephalus scirpaceus, Moustached A-melanopogon and Cetti's Warblers *Cettia cetti* at a Mediterranean stopover site. Ardea 93: 245-257.

Barbraud C, Weimerskirch H (2003) Climate and density shape population dynamics of a marine top predator. Proc Roy Soc Lond B 270: 2111-2116.

Barbraud C, Weimerskirch H, Guinet C, Jouventin P (2000) Effect of sea-ice extent on adult survival of an Antarctic top predator: the snow petrel *Pagodroma nivea*. Oecol 125: 483-488.

Belda EJ, Barba E, Monros JS (2007) Resident and transient dynamics, site fidelity and survival in wintering Blackcaps *Sylvia atricapilla*: evidence from capture-recapture analyses. Ibis 149: 396-404.

Bokony V, Liker A, Lendvai AZ, Kulcsár A (2008) Risk-taking and survival in the House Sparrow *Passer domesticus*: are plumage ornaments costly? Ibis 150: 139-151.

Cam E, Oro D, Pradel R, Jimenez J (2004) Assessment of hypotheses about dispersal in a long-lived seabird using multistate capture-recapture models. J Anim Ecol 73: 723-736.

Clobert J (1995) Capture-recapture and evolutionary ecology: A difficult wedding? J Appl Stat 22: 989-1008.

Clucas RJ, Fletcher DJ, Moller H (2008) Estimates of adult survival rate for three colonies of Sooty Shearwater (*Puffinus griseus*) in New Zealand. Emu 108: 237-250.

Crespin L, Harris MP, Lebreton JD, Wanless S (2006) Increased adult mortality and reduced breeding success with age in a population of common guillemot *Uria aalge* using marked birds of unknown age. J Avian Biol 37: 273-282.

Erikstad KE, Sandvik H, Fauchald P, Tveraa T (2009) Short- and long-term consequences of reproductive decisions: an experimental study in the puffin. Ecology 90: 3197-3208.

Evans RJ, Wilson JD, Amar A, Douse A, Maclennan A, et al. (2009) Growth and demography of a re-introduced population of White-tailed Eagles *Haliaeetus albicilla*. Ibis 151: 244-254.

Forero MG, Tella JL, Oro D (2001) Annual survival rates of adult Red-necked Nightjars *Caprimulgus ruficollis*. Ibis 143: 273-277.

Frederiksen M, Wanless S, Harris MP, Rothery P, Wilson LJ (2004) The role of industrial fisheries and oceanographic change in the decline of North Sea black-legged kittiwakes. J Appl Ecol 41: 1129-1139.

Gauthier, G, Pradel R, Menu S, Lebreton JD (2001) Seasonal survival of Greater Snow Geese and effect of hunting under dependence in sighting probability. Ecology 82: 3105-3119.

Grosbois V, Harris MP, Anker-Nilssen T, McCleery RH, Shaw DN, et al. (2009) Modeling survival at multi-population scales using mark-recapture data. Ecology 90: 2922-2932.

Grosbois V, Thompson PM (2005) North Atlantic climate variation influences survival in adult fulmars. Oikos 109: 273-290.

Hario M, Mazerolle MJ, Saurola PS (2009) Survival of female common eiders *Somateria m. mollissima* in a declining population of the northern Baltic Sea. Oecol 159: 747-756.

Harris, M. P, Anker-Nilssen T, Mccleery RH, Erikstad KE, Shaw DN, et al. (2005) Effect of wintering area and climate on the survival of adult Atlantic puffins *Fratercula arctica* in the eastern Atlantic. Mar Ecol Progr Ser 297: 283-296.

Harris MP, Freeman SN, Wanless S, Morgan BJT, Wernham CV (1997) Factors influencing the survival of Puffins *Fratercula arctica* at a North Sea colony over a 20-year period. J Avian Biol 28: 287-295.

Jenouvrier S, Barbraud C, Weimerskirch H (2003) Effects of climate variability on the temporal population dynamics of southern fulmars. J Anim Ecol 72: 576-587.

Jenouvrier S, Barbraud C, Weimerskirch H (2005) Long-term contrasted responses to climate of two Antarctic seabird species. Ecology 86: 2889-2903.

Jenouvrier S, Thibault J C, Viallefont A, Vidals P, Ristow D et al. (2009) Global climate patterns explain range-wide synchronicity in survival of a migratory seabird. Glob Ch Biol 15: 268-279.

Kaiser A (1995) Estimating turnover, movements and capture parameters of resting passerines in standardized capture-recapture studies. J Appl Stat 22: 1039-1047.

Kauffman MJ, Frick WF, Linthicum J (2003) Estimation of habitat-specific demography and population growth for peregrine falcons in California. Ecol Appl 13: 1802-1816.

Kendall WL, Nichols JD (1995) On the use of secondary capture-recapture samples to estimate temporary emigration and breeding proportions. J Appl Stat 22: 751-762.

Kery M, Madsen J, Lebreton JD (2006) Survival of Svalbard pink-footed geese *Anser brachyrhynchus* in relation to winter climate, density and land-use. J Anim Ecol 75: 1172-1181.

Madsen J, Frederiksen M, Ganter B (2002) Trends in annual and seasonal survival of Pink-footed Geese *Anser brachyrhynchus*. Ibis 144: 218-226.

Monticelli D, Ramos JA, Guerreiro-Milheiras SA, Doucet JL (2008) Adult survival of Tropical Roseate Terns breeding on Aride Island, Seychelles, Western Indian Ocean. Waterbirds 31: 330-337.

Nevoux M, Barbraud C (2006) Relationships between sea ice concentration, sea surface temperature and demographic traits of thin-billed prions. Polar Biol 29: 445-453.

Nevoux M, Weimerskirch H, Barbraud C (2010) Long- and short-term influence of environment on recruitment in a species with highly delayed maturity. Oecol 162: 383-392.

Oro D, De Leon A, Minguez E, Furness RW (2005) Estimating predation on breeding European storm-petrels (*Hydrobates pelagicus*) by yellow-legged gulls *(Larus Michahellis*). J Zool 265: 421-429.

Peron G, Crochet PA, Choquet R, Pradel R, Lebreton JD, et al. (2010) Capture-recapture models with heterogeneity to study survival senescence in the wild. Oikos 119: 524-532.

Pons J, Migot M (1995) Life-history strategy of The Herring Gull - changes in survival and fecundity in a population subjected to various feeding conditions. J Anim Ecol 64: 592-599.

Pradel R, Rioux N, Tamisier A, Lebreton JD (1997) Individual turnover among wintering teal in Camargue: a mark-recapture study. J Wildl Manage 61: 816-821.

Pugesek BH, Nations C, Diem KL, Pradel R (1995) Mark-resighting analysis of a California gull population. J Appl Stat 22: 625-639.

Ratcliffe N, Newton S, Morrison P, Merne O, Cadwallender T, et al. (2008) Adult survival and breeding dispersal of Roseate Terns within the Northwest European metapopulation. Waterbirds 31: 320-329.

Rolland V, Barbraud C, Weimerskirch H (2008) Combined effects of fisheries and climate on a migratory long-lived marine predator. J Appl Ecol 45: 4-13.

Rolland V, Barbraud C, Weimerskirch H (2009) Assessing the impact of fisheries, climate and disease on the dynamics of the Indian yellow-nosed Albatross. Biol Cons 142: 1084-1095.

Rolland V, Nevoux M, Barbraud C, Weimerskirch H (2009) Respective impact of climate and fisheries on the growth of an albatross population. Ecol App 19: 1336-1346.

Sandvik, H, Erikstad K E, Barrett R T, Yoccoz N G (2005) The effect of climate on adult survival in five species of North Atlantic seabirds. J Anim Ecol 74: 817-831.

Sandvik H, Erikstad KE, Fauchald P, Tveraa T (2008) High survival of immatures in a long-lived seabird: Insights from a long-term study of the Atlantic Puffin (*Fratercula arctica*). Auk 125: 723-730.

Sanz-Aguilar A, Tavecchia G, Mínguez E, Massa B, Lo Valvo F, et al. (2010) Recapture processes and biological inference in monitoring burrowing nesting seabirds. J Ornithol 151: 133-146.

Sanz-Aguilar A, Tavecchia G, Genovart M, Igual JM, Oro D, et al. (2011) Studying the reproductive skipping behavior in long-lived birds by adding nest-inspection to individual-based data. Ecol Appl 21: 555-564.

Schaub M, Kania W, Koppen U (2005) Variation of primary production during winter induces synchrony in survival rates in migratory white storks *Ciconia ciconia*. J Anim Ecol 74: 656-666.

Spendelow JA , Nichols JD, Hines JE, Lebreton JD, Pradel R (2002) Modelling postfledging survival and age-specific c breeding probabilities in species with delayed maturity: a case study of Roseate Terns at Falkner Island, Connecticut. J Appl Stat, 29: 385-405.

Szep T (1999) Effects of age- and sex-biased dispersal on the estimation of survival rates of the Sand Martin *Riparia riparia* population in Hungary. Bird Stu 46: 169-177.

Tavecchia G, Minguez E, De León A, Louzao M, Oro D (2008) Living close, doing differently: Small-scale asynchrony in demography of two species of seabirds. Ecology 89: 77-85.

Tavecchia G, Viedma C, Martínez-Abraín A, Bartolomé MA, Gómez JA, et al. (2009) Maximizing re-introduction success: Assessing the immediate cost of release in a threatened waterfowl. Biological Conservation, 142, 3005-3012.

Viallefont A, Cooch EG, Cooke F (1995) Estimation of trade-offs with capture-recapture models: A case study on the lesser snow goose. J Appl Stat 22: 847-861.

Viallefont A, Cooke F, Lebreton JD (1995) Age-specific costs of first-time breeding. Auk 112: 67-76.

Votier SC, Hatchwell BJ, Beckerman A, McCleery RH, Hunter FM, et al. (2005) Oil pollution and climate have wide-scale impacts on seabird demographics. Ecol Lett 8: 1157-1164.

Bird studies not correcting for trap-dependence

Bearhop S, Ward RM, Evans PR (2003) Long-term survival rates in colour-ringed shorebirds - practical considerations in the application of mark-recapture models. Bird Stu 50: 271-279.

Cooch, E. G, Blank DB, Rockwell RF, Cooke F (1999) Body size and age of recruitment in Snow Geese *Anser c. caerulescens*. Bird Stu 46: 112-119.

Dugger, K. M, Ainley DG, Lyver POB, Barton K, Ballardef G (2010) Survival differences and the effect of environmental instability on breeding dispersal in an Adelie penguin meta-population. Proc Natl Acad Sci USA 107: 12375-12380.

Faustino, C.R, Jennelle CS, Connolly V, Davis AK, Swarthout EC, et al. (2004) *Mycoplasma gallisepticum* infection dynamics in a house finch population: seasonal variation in survival, encounter and transmission rate. J Anim Ecol 73: 651-669.

Frederiksen M, Bregnballe T (2000) Evidence for density-dependent survival in adult cormorants from a combined analysis of recoveries and resightings. J Anim Ecol 69: 737-752.

Frederiksen M, Bregnballe T (2001) Conspecific reproductive success affects age of recruitment in a great cormorant, *Phalacrocorax carbo sinensis*, colony. Proc Roy Soc Lond B 268: 1519-1526.

Lebreton, J. D, Hines JE, Pradel R, Nichols JD, Spendelow JA (2003) Estimation by capture-recapture of recruitment and dispersal over several sites. Oikos 101: 253-264.

Peach WJ, Hanmer DB, Oatley TB (2001) Do southern African songbirds live longer than their European counterparts? Oikos 93: 235-249.

Schaub M, Jenni L (2001) Stopover durations of three warbler species along their autumn migration route. Oecol 128: 217-227.

Votier SC, Birkhead TR, Oro D, Trinder M, Grantham MJ, et al. (2008) Recruitment and survival of immature seabirds in relation to oil spills and climate variability. J Anim Ecol 77: 974-983.

Mammal studies correcting for trap-dependence

Crespin L, Choquet R, Lima M, Merritt J, Pradel R (2008) Is heterogeneity of catchability in capture-recapture studies a mere sampling artefact or a relevant feature of the population? Pop Ecol 50: 247-256.

Crespin L, Papillon Y, Abdoulaye D, Granjon L, Sicard B (2008) Annual flooding, survival and recruitment in a rodent population from the Niger River plain in Mali. J Trop Ecol 24: 375-386.

Guitton JS, Devillard S, Guénézan M, Fouchet D, Pontier D, et al. (2008) Vaccination of free-living juvenile wild rabbits (*Oryctolagus cuniculus*) against myxomatosis improved their survival. Prev Vet Med 84: 1-10.

Hoyle SD, Pople AR, Toop GJ (2001) Mark-recapture may reveal more about ecology than about population trends: Demography of a threatened ghost bat (*Macroderma gigas*) population. Austral Ecol 26: 80-92.

Johannesen E, Aars J, Andreassen HP, Ims RA (2003) A demographic analysis of vole population responses to fragmentation and destruction of habitat. Pop Ecol 45: 47-58.

Julliard R, Leirs H, Stenseth NC, Yoccoz NG, Prevot-Julliard AC, et al. (1999) Survival-variation within and between functional categories of the African multimammate rat. J Anim Ecol 68: 550-561.

1Kraus C, Eberle M, Kappeler PM (2008) The costs of risky male behaviour: sex differences in seasonal survival in a small sexually monomorphic primate. Proc Roy Soc Lond B 275: 1635-1644.

Langtimm CA, O’Shea TJ, Pradel R, Beck CA (1998) Estimates of annual survival probabilities for adult Florida manatees (*Trichechus manatus latirostris*). Ecology 79: 981-997.

Largo E, Gaillard JM, Festa-Bianchet M Toïgo C, Bassano B, et al. (2008) Can ground counts reliably monitor ibex *Capra ibex* populations? Wildl Biol 14: 489-499.

Letty J, Ubineau JA, Marchandeau S, Lobert JC (2003) Effect of translocation on survival in wild rabbit (*Oryctolagus cuniculus*). Mammal Biol 68: 250-255.

Lima M, Merritt JF, Bozinovic F (2002) Numerical fluctuations in the northern short-tailed shrew: evidence of non-linear feedback signatures on population dynamics and demography. J Anim Ecol 71: 159-172.

Pendleton GW, Pitcher KW, Fritz L, Raum-Suryan KL, Loughlin T, et al. (2006) Survival of Steller sea lions in Alaska: a comparison of increasing and decreasing populations. Cn J Zool 84: 1163-1172.

Pradel R, Choquet R, Lima MA, Merritt J, Crespin L (2010) Estimating population growth rate from capture-recapture data in presence of capture heterogeneity. J Agrt Biol Environ Stat 15: 248-258.

Ramp, C. Bérubé M, Hagen W, Sears R (2006) Survival of adult blue whales *Balaenoptera musculus* in the Gulf of St. Lawrence, Canada. Mar Ecol Progr Ser 319: 287-295.

Ramp C, Bérubé M, Palsbøll P, Hagen W, Sears R (2010) Sex-specific survival in the humpback whale *Megaptera novaeangliae* in the Gulf of St. Lawrence, Canada. Mar Ecol Progr Ser 400: 267-276.

Regehr EV, Regehr EV, Lunn N, Amstrup SC, Stirling I (2007) Effects of earlier sea ice breakup on survival and population size of polar bears in western Hudson bay. J Wildl Manage 71: 2673-2683.

Sendor T, Simon M (2003) Population dynamics of the pipistrelle bat: effects of sex, age and winter weather on seasonal survival. J Anim Ecol 72: 308-320.

Telfer S, Bennett M, Bown K, Cavanagh R, Crespin Laurent, et al. (2002) The effects of cowpox virus on survival in natural rodent populations: increases and decreases. J Anim Ecol 71: 558-568.

Mammal studies not correcting for trap-dependence

Beauplet G, Barbraud C, Dabin W, Küssener C, Guinet C (2006) Age-specific survival and reproductive performances in fur seals: evidence of senescence and individual quality. Oikos 112/ 430-441.

Graham IM, Lambin X (2002) The impact of weasel predation on cyclic field-vole survival: the specialist predator hypothesis contradicted. J Anim Ecol 71: 946-956.

Schwarz CJ, Stobo WT (2000) Estimation of juvenile survival, adult survival, and age-specific pupping probabilities for the female grey seal (*Halichoerus gryprus*) on Sable Island from capture-recapture data. Can J Fish Aqu Sci 57: 247-253.

Sluydts V, Crespin L, Davis S, Lima M, Leirs H (2007) Survival and maturation rates of the African rodent, Mastomys natalensis: density-dependence and rainfall. Integr Zool 2/ 220-232.

Reptile studies correcting for trap-dependence

Rivalan P, Pradel R, Choquet R, Girondot M, Prévot-Julliard AC (2006) Estimating clutch frequency in the sea turtle Dermochelys coriacea using stopover duration. Mar Ecol Progr Ser 317: 285-295.

Rivalan P, Prévot-Julliard AC, Choquet R, Pradel R, Jacquemin B, et al. (2005) Trade-off between current reproductive effort and delay to next reproduction in the leatherback sea turtle. Oecol 145: 564-574.

Reptile studies not correcting for trap-dependence

Bjorndal KA, Bolten AB, Chaloupka MY (2003) Survival probability estimates for immature green turtles *Chelonia mydas* in the Bahamas. Mar Ecol Progr Ser 252: 273-281.

Bjorndal KA, Bolten AB, Chaloupka MY (2005) Evaluating trends in abundance of immature green turtles, *Chelonia mydas*, in the Greater Caribbean. Ecol Appl 15: 304-314.

Chaloupka MY, Limpus CJ (2002) Survival probability estimates for the endangered loggerhead sea turtle resident in southern Great Barrier Reef waters. Mar Biol 140: 267-277.

Amphibian studies correcting for trap-dependence

Fretey T, Cam E, Le Garff B, Monnat JY (2004) Adult survival and temporary emigration in the common toad. Can J Zool 82: 859-872.

Schmidt BR, Schaub M, Anholt BR (2002) Why you should use capture-recapture methods when estimating survival and breeding probabilities: on bias, temporary emigration, overdispersion, and common toads. Amph-Rept 23: 375-388.

Fish studies correcting for trap-dependence

Wormald CL, Steele MA (2008) Testing assumptions of mark-recapture theory in the coral reef fish *Lutjanus apodus*. J Fish Biol 73: 498-509.

Fish studies not correcting for trap-dependence

Holmberg J, Norman B, Arzoumanian Z (2008) Robust, comparable population metrics through collaborative photo-monitoring of whale sharks *Rhincodon Typus*. Ecol Appl 18: 222-233.

Insect studies correcting for trap-dependence

Stoks R (2001) What causes male-biased sex ratios in mature damselfly populations? Ecol Entomol 26: 188-197.
