# Supplementary material for: Modeling Trap-Awareness and Related Phenomena in Capture-Recapture Studies
Source: PLoS One. 2012 Mar 2;7(3):e32666. doi: 10.1371/journal.pone.0032666 (PMC3292565; doi:10.1371/journal.pone.0032666)
Supplement: Appendix S2 — Practical implementation of multievent trap-dependence models with program E-SURGE: a medium-term monitoring program on Cory's Shearwater (Calonectris diomedea) as a case example. (DOCX) [file pone.0032666.s002.docx]

**Appendix S2**

**Practical implementation of multievent trap-dependence models with program** [**E-SURGE**](http://www.cefe.cnrs.fr/BIOM/logiciels.htm)**: a medium-term monitoring program on Cory’s Shearwater (*Calonectris diomedea*) as a case example.**

The Cory’s shearwater (*Calonectris diomedea*) is a long-lived burrow nesting seabird. Skipping reproduction is typical among them and usually confounded with a recapture failure. Because skippers in one year have a higher probability of skipping the following year, trap-dependence (*lato sensu*) is usually found when analyzing Cory’s Shearwater capture histories. Sanz-Aguilar et al. (2011) combined individual-based information with nest-based information collected as part of the Cory’s Shearwater monitoring program of the [Population Ecology Group](http://www.imedea.uib.es/bc/gep/) (IMEDEA, Esporles, Spain) carried out at Pantaleu Island (Balearic Islands, Spain). Using multievent models, they estimated simultaneously recapture, survival, reproductive skipping and within-colony breeding dispersal probabilities. Here, for the sake of illustrating how to treat trap-dependence in different contexts, we consider simpler and more typical situations which use only part of the available information.

1. We start with the situation described in the first section of the methods, i.e. we keep only the information about whether the individual is ‘seen’ (code 1) or ‘not seen’ (code 0), and we show how to fit the single state model with trap-dependence allowing the estimation of survival and capture probabilities.
2. We next illustrate the second section of the methods by considering 2 observable states: the bird occupies its ‘last known burrow’ (code 1) or the bird occupies a ‘new burrow’ (code 2). We show how to fit a multistate model with trap-dependence allowing the estimation of survival, capture probabilities and the probability of burrow change.
3. Finally, we consider a situation with two ambiguous events relative to the underlying state: the classical event ‘not seen with no further information’ (code 0) and the more informative but still imperfect event ‘not seen but known to be absent from its previous burrow’ (previous burrow is empty or occupied by others) (code 2). Code 1 is kept as usual for the event ‘seen’. We show how to fit this pure multievent model with trap dependence to estimate survival, capture probabilities and the probability of knowing that the bird is absent from its previous burrow. This model has no more interest than showing the basics of how trap-dependence and ambiguous events can be combined. For a fuller exploitation of burrow information, we refer the reader to Sanz-Aguilar et al. (2011).

**Specification of the multievent modelling approach in program E-SURGE** (extracted from Sanz-Aguilar et al. 2011)

Multievent models are built in several stages using program E-SURGE (Choquet et al. 2009). Each step represents one type of the different parameters to estimate. This is done by means of row-stochastic matrices, i.e. each row corresponds to a multinomial. Consequently, the total of cell probabilities in the same row is 1. Because of this constraint, one and only one cell probability in each row will be calculated as the complement to 1 of the others. This particular cell is denoted with a ‘*’ symbol. Inactive cells, i.e. cells whose associated probability is structurally 0 are denoted with a ‘-’ symbol. An active cell receives an arbitrary letter. Note that the same letter in two cells *does not mean* that the two values should be equal.

1. **Estimating survival and recapture probabilities.**

The individual states considered are:

**A**, “trap-aware”

**U**, “trap-unaware”

**D**, dead

The possible events are:

**0**, not recaptured

**1**, captured or recaptured

The symbols for parameters are:

**φ**, survival probability

**p**, capture probability

Initial State probabilities

| ***A*** | ***U*** |
| --- | --- |
| ******* | ***-*** |

Transition probabilities, step 1: Survival

|  | ***A _t+1_^-^*** | ***U _t+1_^-^*** | ***D _t+1_^-^*** |
| --- | --- | --- | --- |
| ***A _t_*** | **φ** | **-** | ***** |
| ***U _t_*** | **-** | **φ** | ***** |

Transition probabilities, step 2: Trap awareness process

|  | ***A _t+1_*** | ***U _t+1_*** | ***D _t+1_*** |
| --- | --- | --- | --- |
| ***A _t+1_^-^*** | **p** | ***** | **-** |
| ***U _t+1_^-^*** | **p** | ***** | **-** |
| ***D _t+1_^-^*** | **-** | **-** | ***** |

Event probabilities:

|  | ***0*** | ***1*** |
| --- | --- | --- |
| ***A_t_*** | **-** | ***** |
| ***U _t_*** | ***** | **-** |
| ***D _t_*** | ***** | **-** |

***Detailed example of fitting model 5, Table 2 of Sanz-aguilar et al. (2011) with program E-SURGE***

*After reading the data into program E-SURGE, the number of states is changed to 3 and the number of age-classes is changed to 2 (2 age-classes are needed to account for the presence of transients in this data set, see Pradel et al. 1997).*

*
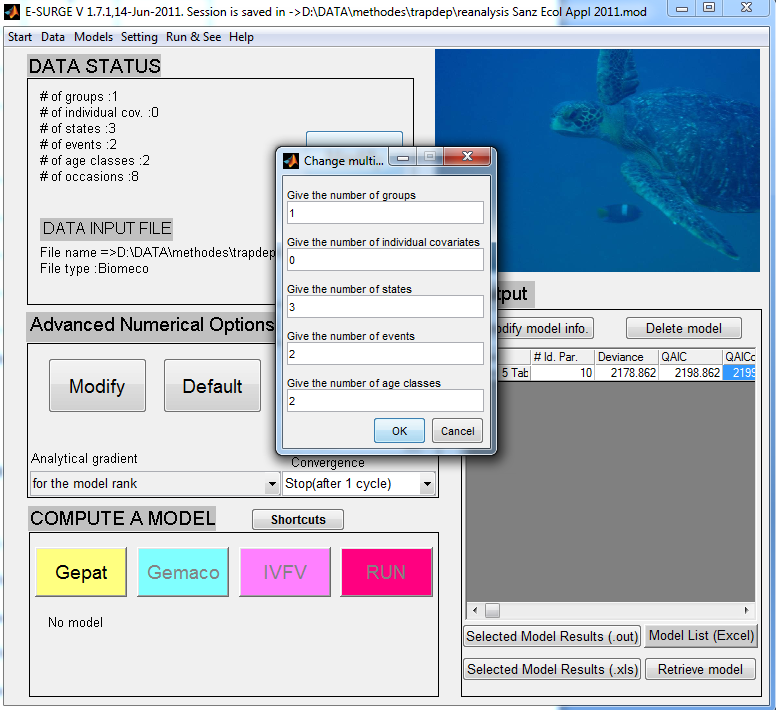
*

*Then, enter the GEPAT interface to specify the patterns as we have seen above: first, for the initial state probabilities,*

*
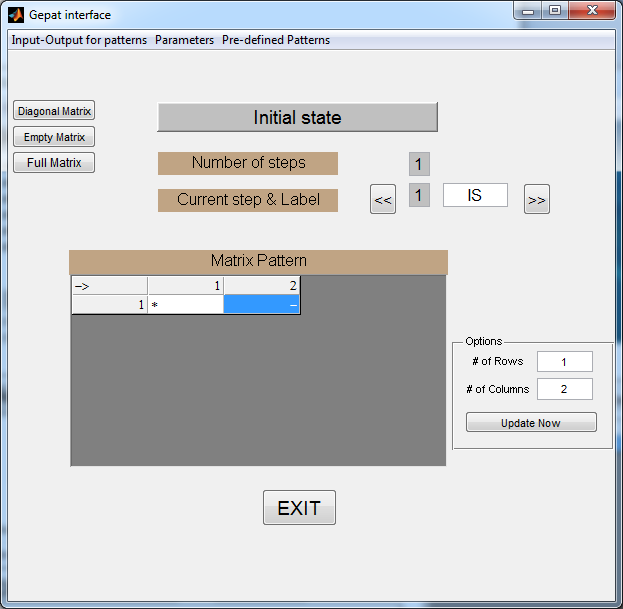
*

*then for the transitions for which 2 steps must be specified.*

*
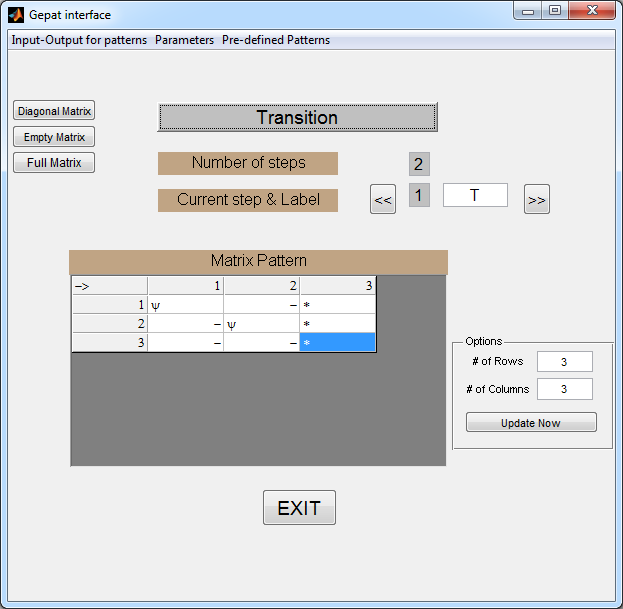

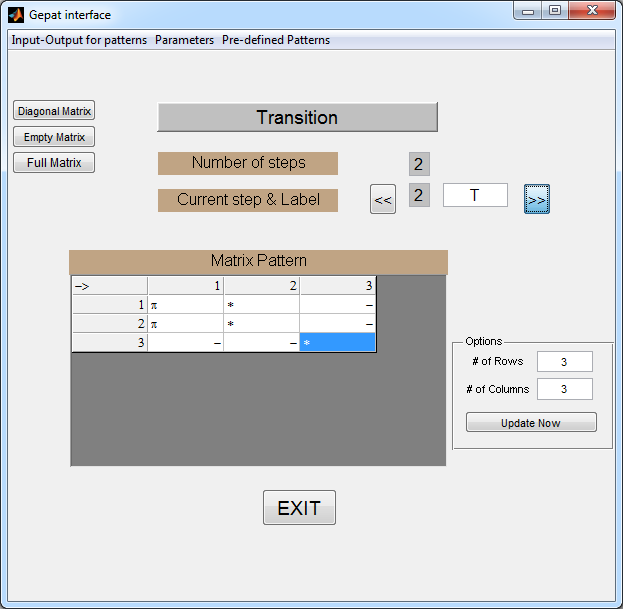
*

*and eventually for the events.*

*
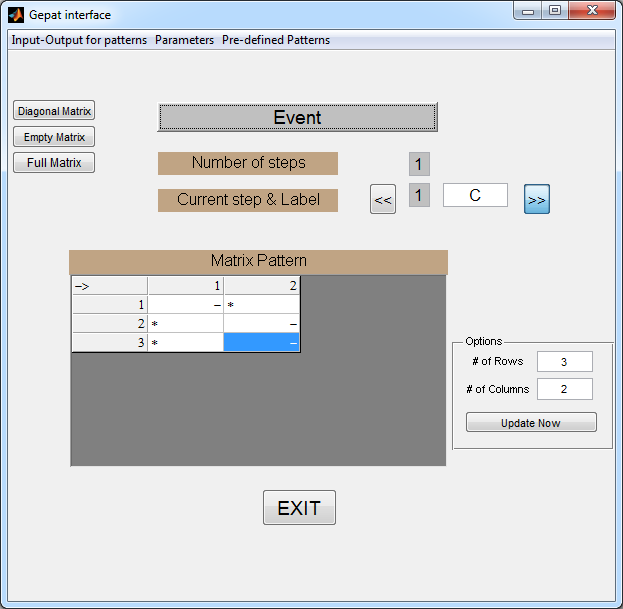
*

*After that, click EXIT and, back on the main window, GEMACO to enter the GEMACO interface where effects are specified on each type of parameter in turn.*

*For the initial state probability, there is no active parameter. The keyword* ‘i’ *will do.*

*
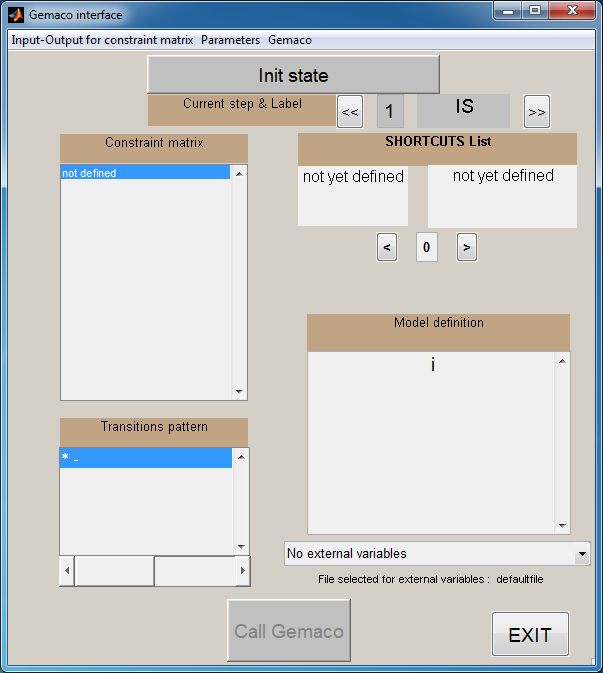
*

*The first step of transitions corresponds to survival probabilities which depend only on age: keyword* ‘a’*.*

*
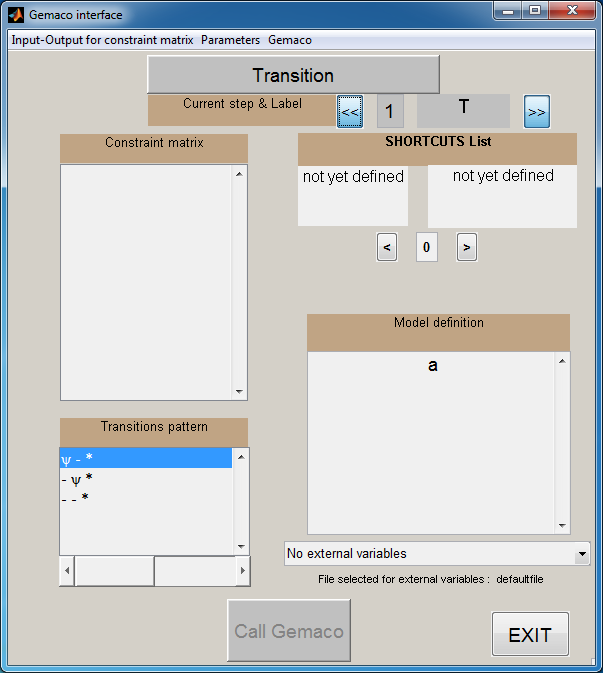
*

*The second step involves capture probabilities, which in this model depend on the trap-awareness status and on time, the two effects being additive: phrase* ‘f+t’*.* (‘f’ *short for* ‘from’ *means that there is a row effect, which here is the trap-awareness status effect).*

*
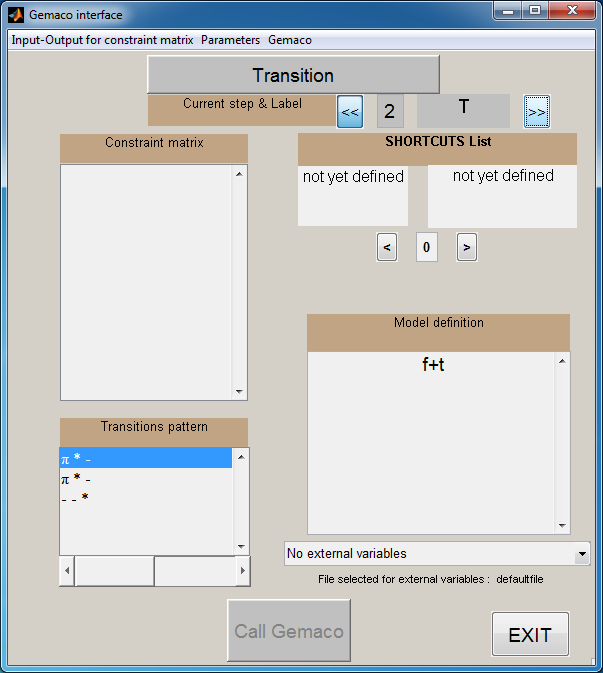
*

*Event probabilities are not active. The keyword* ‘i’ *will do the job.*

*
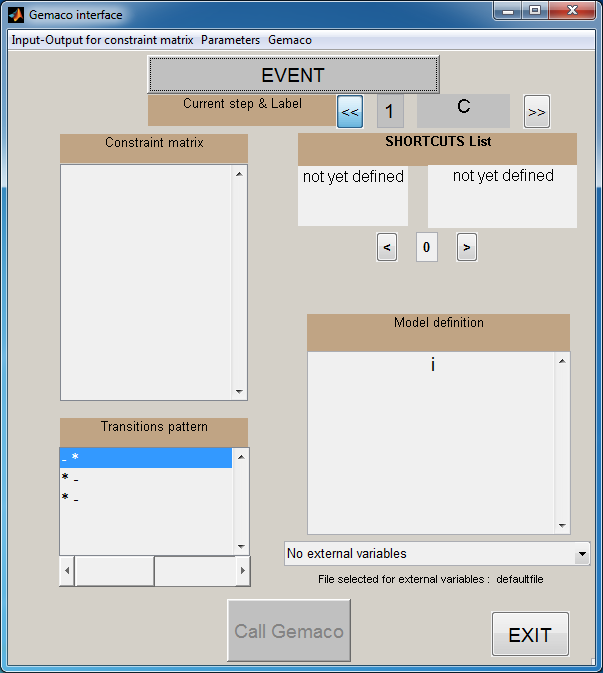
*

*After clicking EXIT, then back in the main windows, IVFV and EXIT again, you can run the model by clicking RUN.*

1. **Estimating survival, nest dispersal and recapture probabilities.**

The individual states considered are:

**A1**, “trap-aware” and breeding in the same nest as in the previous year

**U1**, “trap-unaware” and breeding in the same nest as in the previous year

**A2**, “trap-aware” and breeding in another nest

**U2**, “trap-unaware” and breeding in another nest

**D**, dead

The possible events are:

**0**, not recaptured

**1**, captured for the first time or recaptured breeding in the last known nest

**2**, recaptured breeding in a different nest

The symbols for parameters are:

**φ**, survival probability

**ψ**, nest dispersal probability, conditional on survival

**p**, capture probability

Initial State probabilities

| ***A1*** | ***U1*** | ***A2*** | ***U2*** |
| --- | --- | --- | --- |
| ***π*** | ***-*** | ******* | ***-*** |

Transition probabilities, step 1: Survival

|  | ***A1 _t+1_^-^*** | ***U1 _t+1_^-^*** | ***A2 _t+1_^-^*** | ***U2 _t+1_^-^*** | ***D _t+1_^-^*** |
| --- | --- | --- | --- | --- | --- |
| ***A1 _t_*** | **φ** | **-** | **-** | **-** | ***** |
| ***U1 _t_*** | **-** | **φ** | **-** | **-** | ***** |
| ***A2 _t_*** | **-** | **-** | **φ** | **-** | ***** |
| ***U2 _t_*** | **-** | **-** | **-** | **φ** | ***** |
| ***D _t_*** | **-** | **-** | **-** | **-** | ***** |

Transition probabilities, step 2: Nest dispersal

|  | ***A1 _t+1_^-^*** | ***U1 _t+1_^-^*** | ***A2 _t+1_^-^*** | ***U2 _t+1_^-^*** | ***D _t+1_^-^*** |
| --- | --- | --- | --- | --- | --- |
| ***A1 _t+1_^-^*** | ***** | **-** | **ψ** | **-** | **-** |
| ***U1 _t+1_^-^*** | **-** | ***** | **-** | **ψ** | **-** |
| ***A2 _t+1_^-^*** | ***** | **-** | **ψ** | **-** | **-** |
| ***U2 _t+1_^-^*** | **-** | ***** | **-** | **ψ** | **-** |
| ***D _t+1_^-^*** | **-** | **-** | **-** | **-** | ***** |

Transition probabilities, step 3: Trap awareness process

|  | ***A1 _t+1_*** | ***U1 _t+1_*** | ***A2 _t+1_*** | ***U2 _t+1_*** | ***D _t+1_*** |
| --- | --- | --- | --- | --- | --- |
| ***A1 _t+1_^-^*** | **p** | ***** | **-** | **-** | **-** |
| ***U1 _t+1_^-^*** | **p** | ***** | **-** | **-** | **-** |
| ***A2 _t+1_^-^*** | **-** | **-** | **p** | ***** | **-** |
| ***U2 _t+1_^-^*** | **-** | **-** | **p** | ***** | **-** |
| ***D _t+1_^-^*** | **-** | **-** | **-** | **-** | ***** |

Event probabilities:

|  | ***0*** | ***1*** | ***2*** |
| --- | --- | --- | --- |
| ***A1_t_*** | **-** | ***** | **-** |
| ***U1 _t_*** | ***** | **-** | **-** |
| ***A2 _t_*** | **-** | **-** | ***** |
| ***U2 _t_*** | ***** | **-** | **-** |
| ***D _t_*** | ***** | **-** | **-** |

***Detailed example of fitting a multistate model with trap-dependence with program E-SURGE***

*After reading the data into program E-SURGE, the number of states is changed to 5 and the number of age-classes to 2 (this is to deal with transients). Then we go through GEPAT to specify the patterns as indicated above.*

*
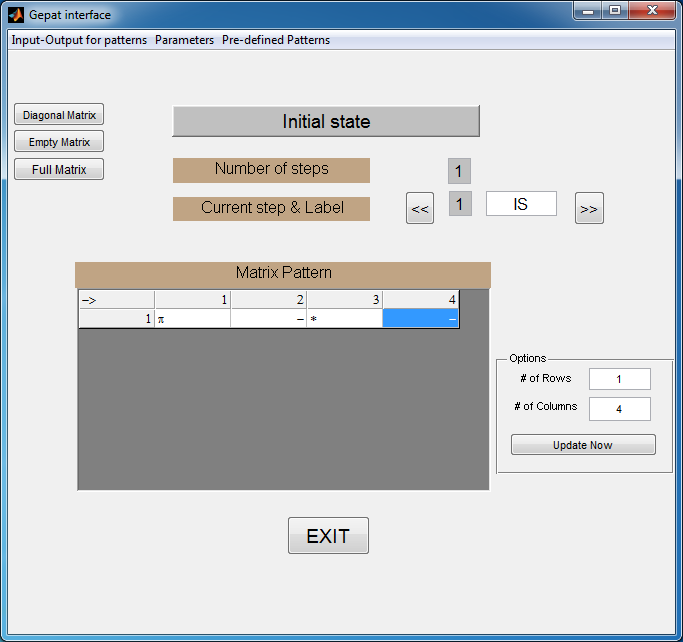
*

*
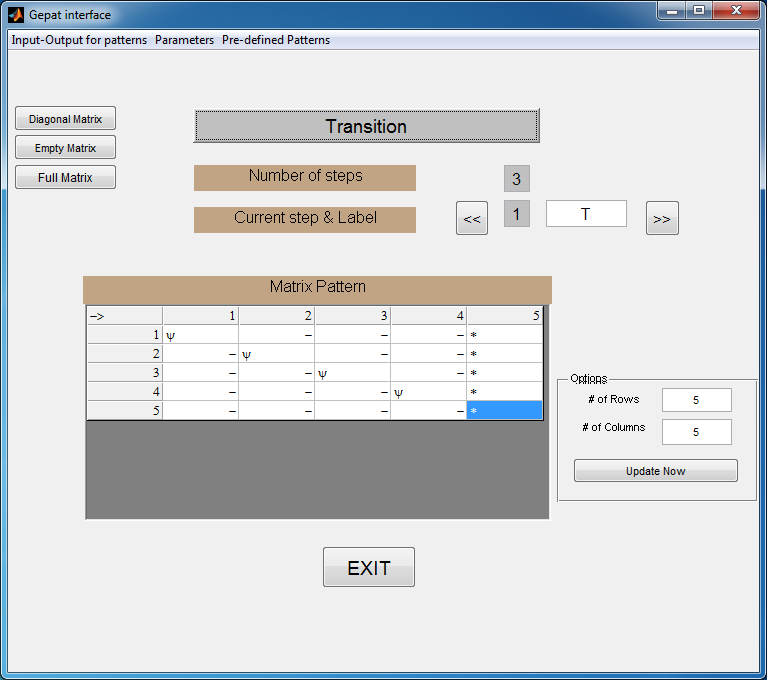
*

*
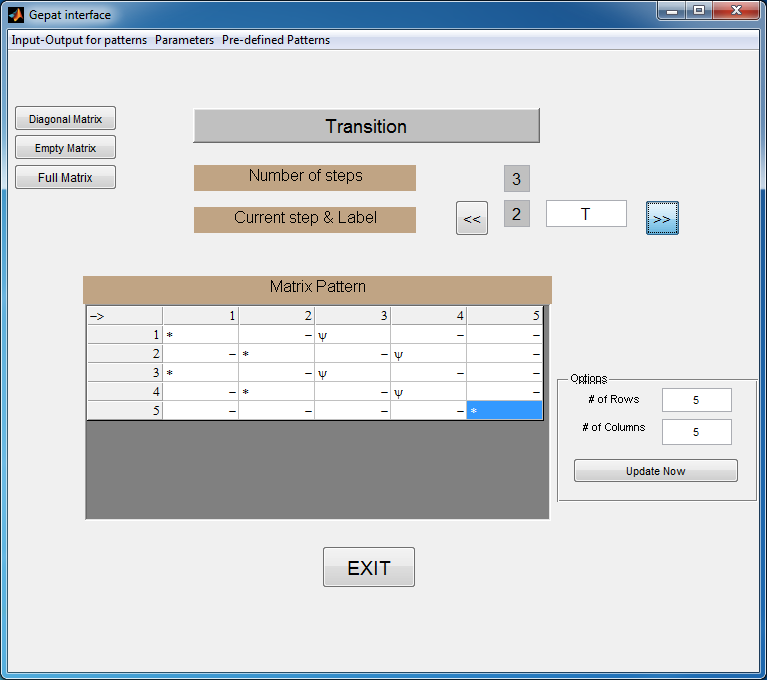
*

*
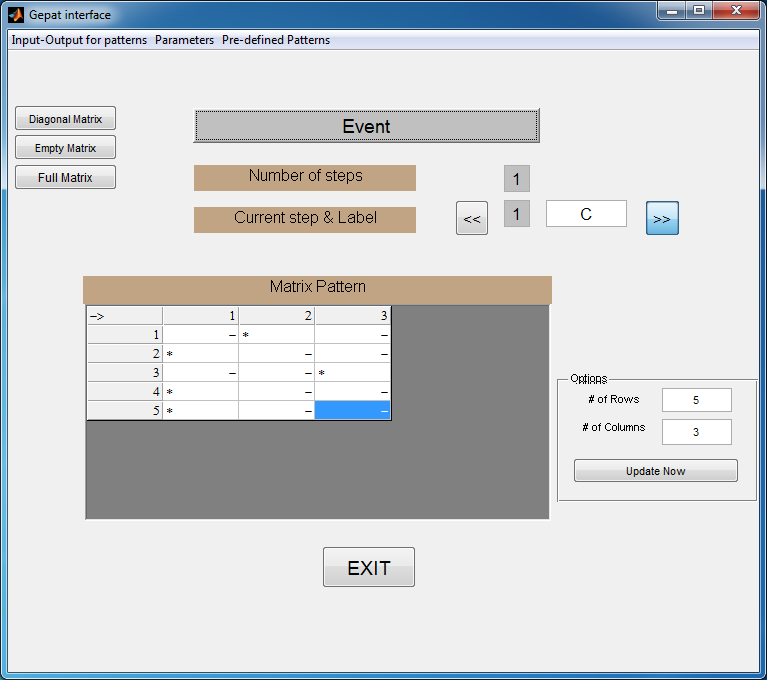
*

*In GEMACO, we specify that the initial state probabilities will be constant using the keyword* ‘i’. *This will suffice because all Cory’s shearwaters are arbitrarily assumed to be in the same nest as the year before when first encountered, i.e. initial state A1. (Alternatively, for this particular data set, we could have entered* $\left( \begin{matrix} * & - & \begin{matrix} - & - \end{matrix} \end{matrix} \right)$ *in GEPAT)*

*
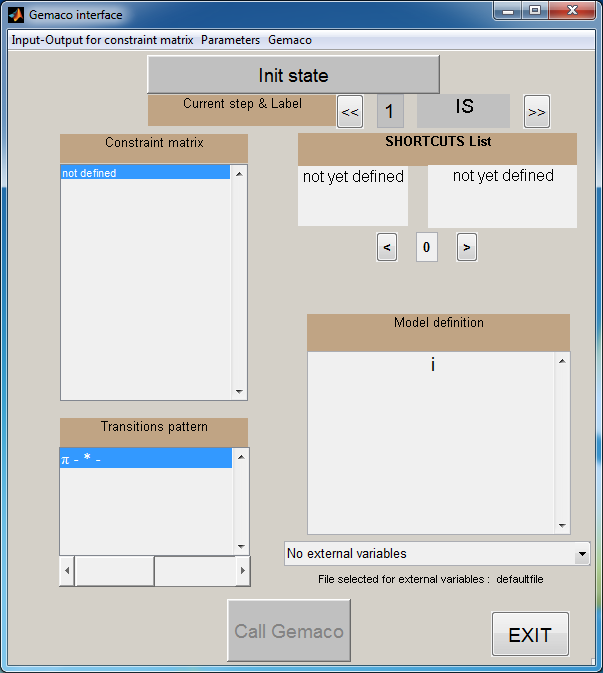
*

*Survival as above is assumed to depend on age to account for the presence of transients.*

*
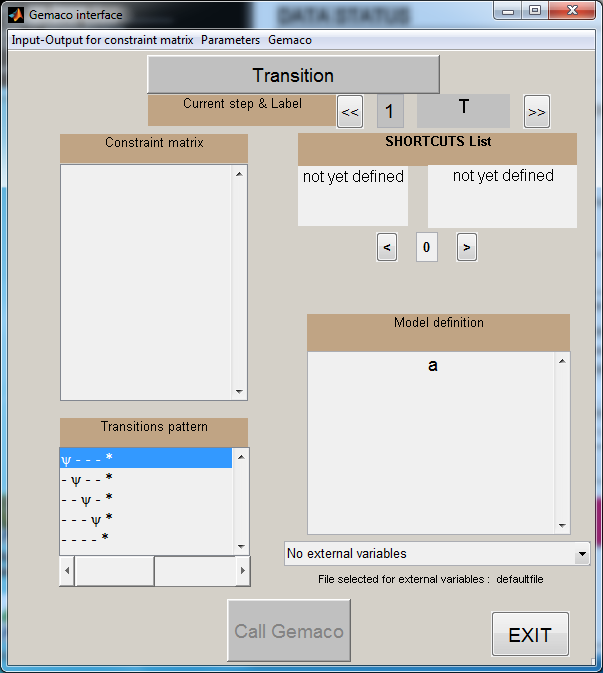
*

*Burrow transition is assumed independent of a previous transition, trap-awareness, etc.: keyword* ‘i’.

*
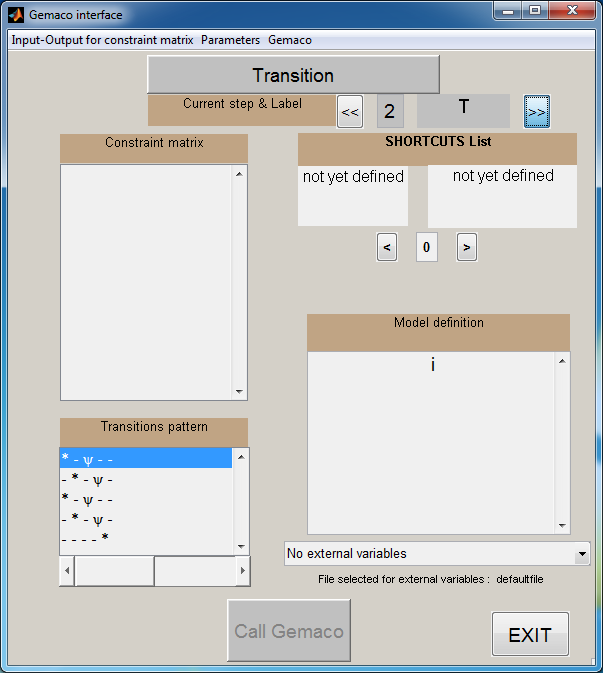
*

*Capture probability depends as before on time and trap-awareness status additively. Because trap-aware individuals are those in the operative states A1 (row 1) and A2 (row 3), the phrase is* ‘t+f(1 3)’.

***
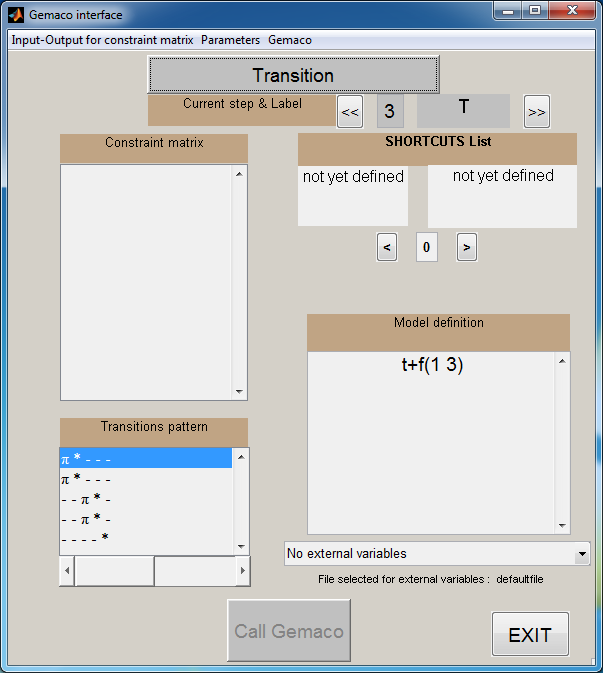
***

*There is no active event probability. This step is as above.*

*
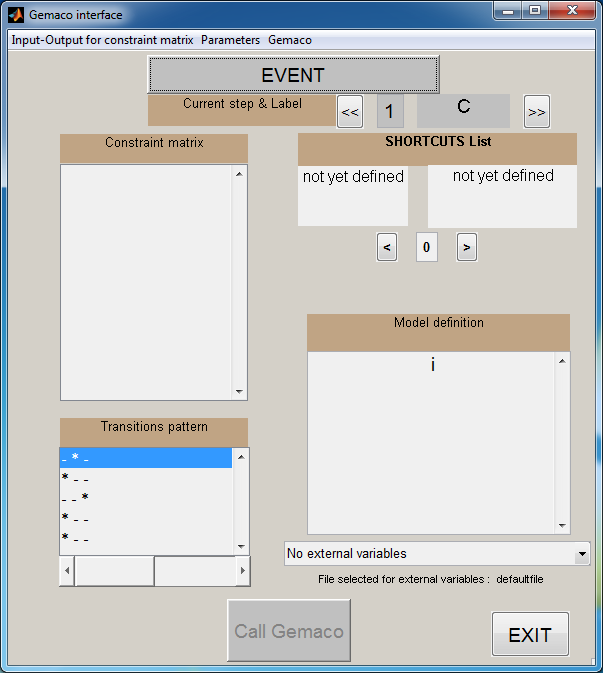
*

*The rest is pretty much unchanged, except that in IVFV it may help to fix the initial state probability to 1 as we know that all individuals start in state A1.*

*
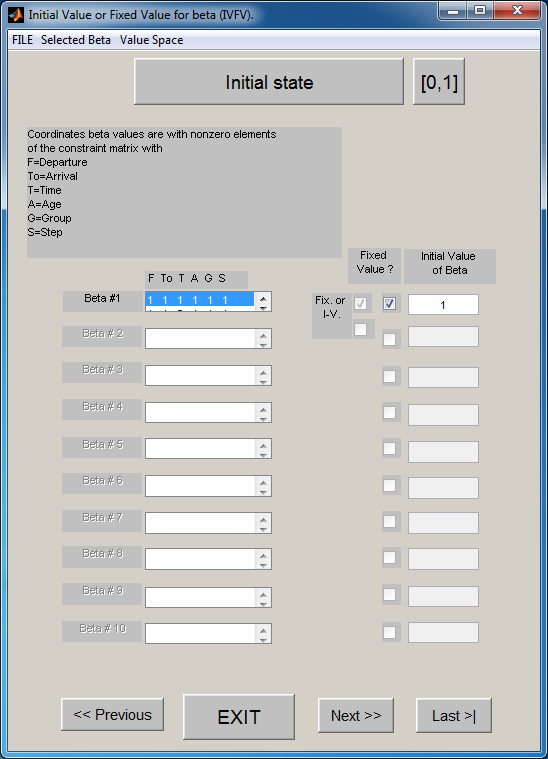
*

1. **Estimating survival and recapture probabilities, and the probability of knowing that a bird is absent from its previous burrow.**

The individual states considered are:

**A**, “trap-aware”

**U**, “trap-unaware”

**D**, dead

The possible events are:

**0**, not recaptured without further information

**1**, captured or recaptured

**2**, not recaptured and known to be absent from its previous burrow (previous burrow is found empty or occupied by others)

The symbols for parameters are:

**φ**, survival probability

**p**, capture probability

**Ɛ**, probability of knowledge of absence from previous burrow

Initial State probabilities

| ***A*** | ***U*** |
| --- | --- |
| ******* | ***-*** |

Transition probabilities, step 1: Survival

|  | ***A _t+1_^-^*** | ***U _t+1_^-^*** | ***D _t+1_^-^*** |
| --- | --- | --- | --- |
| ***A _t_*** | **φ** | **-** | ***** |
| ***U _t_*** | **-** | **φ** | ***** |

Transition probabilities, step 2: Trap awareness process

|  | ***A _t+1_*** | ***U _t+1_*** | ***D _t+1_*** |
| --- | --- | --- | --- |
| ***A _t+1_^-^*** | **p** | ***** | **-** |
| ***U _t+1_^-^*** | **p** | ***** | **-** |
| ***D _t+1_^-^*** | **-** | **-** | ***** |

Event probabilities:

|  | ***0*** | ***1*** | ***2*** |
| --- | --- | --- | --- |
| ***A_t_*** | **-** | ***** | **-** |
| ***U _t_*** | ***** | **-** | **Ɛ** |
| ***D _t_*** | ***** | **-** | **Ɛ** |

***Detailed example of fitting a pure multievent model with trap-dependence with program E-SURGE***

*After reading the data into program E-SURGE, the number of states is changed to 3 and the number of age-classes to 2 (this last point is to account for the presence of transients). Then we go through GEPAT to specify the patterns as indicated above.*


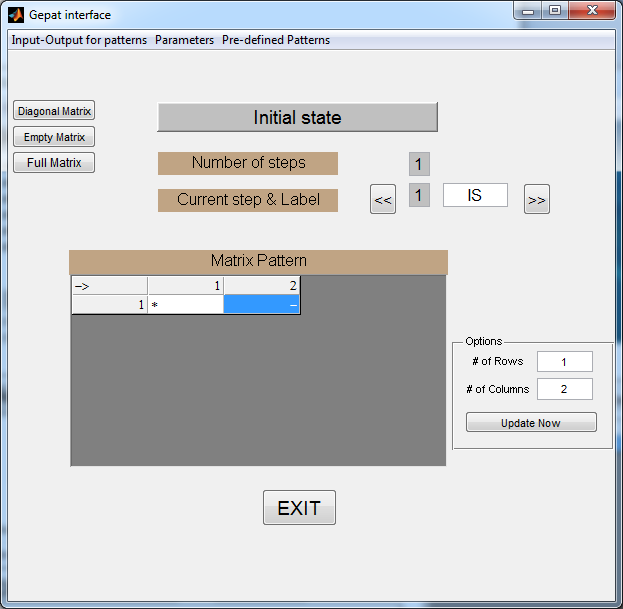


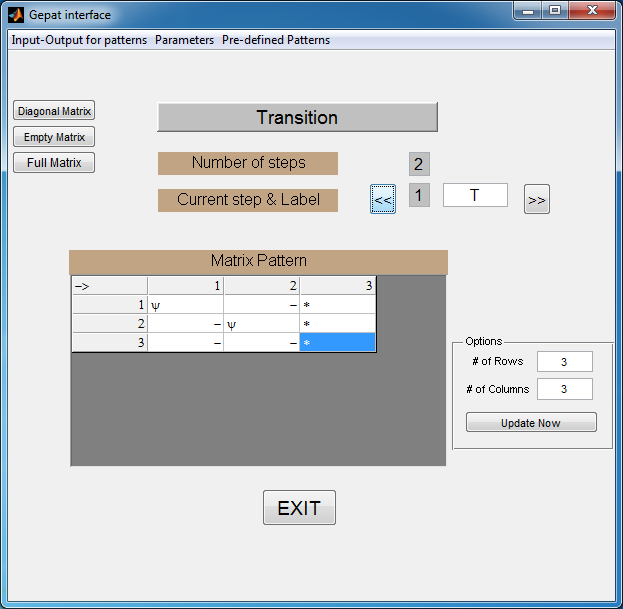


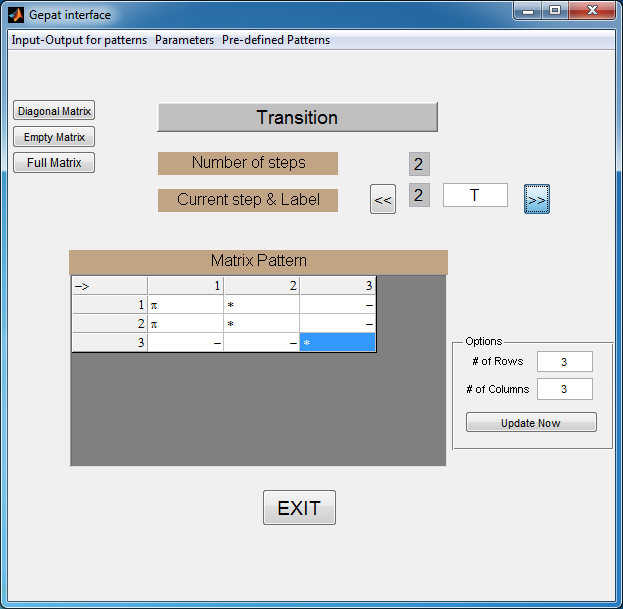


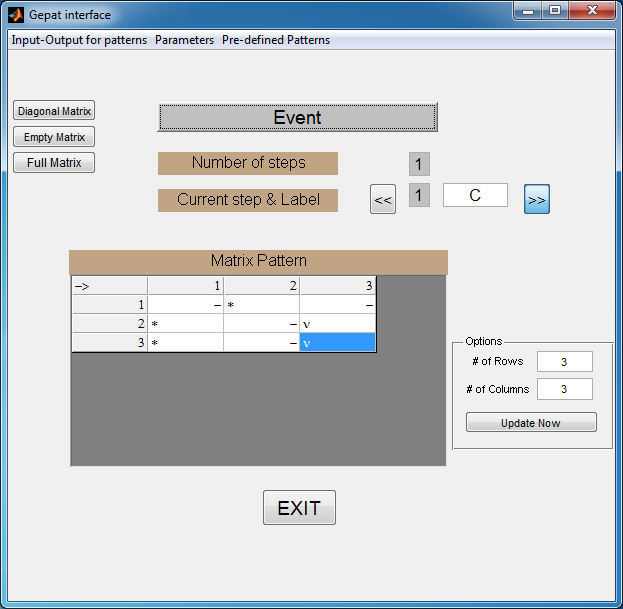


*In GEMACO, as there is no active parameter in the initial state probabilities, we can use the keyword* ‘i’*.*


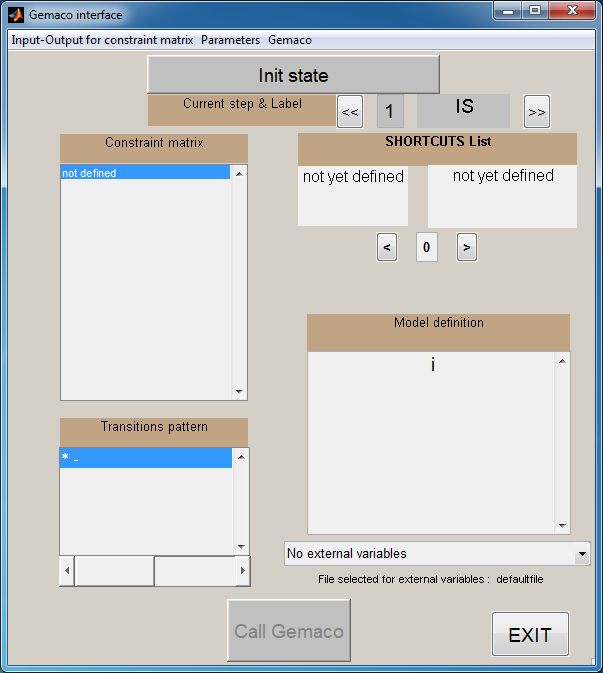


*For the survival probabilities, we need age to account for transients.*
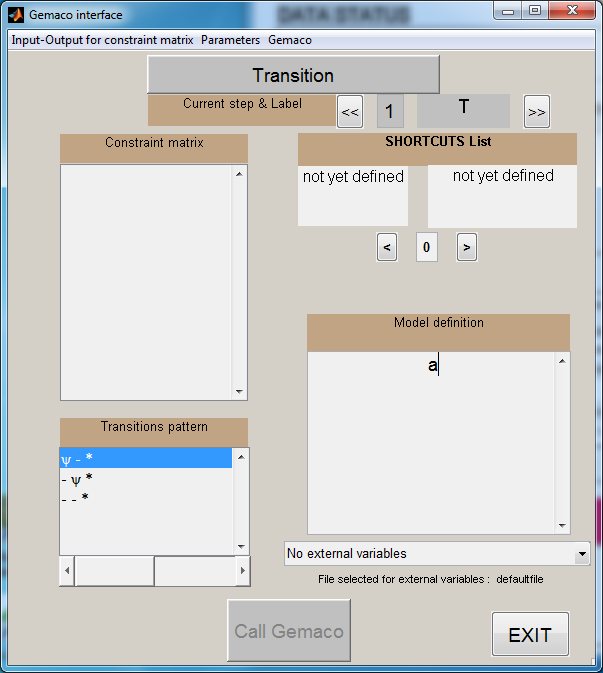


*For capture probabilities, we specify an additive effect of time and trap-awareness status. Here, trap-aware individuals are those in state A1, the first state.*


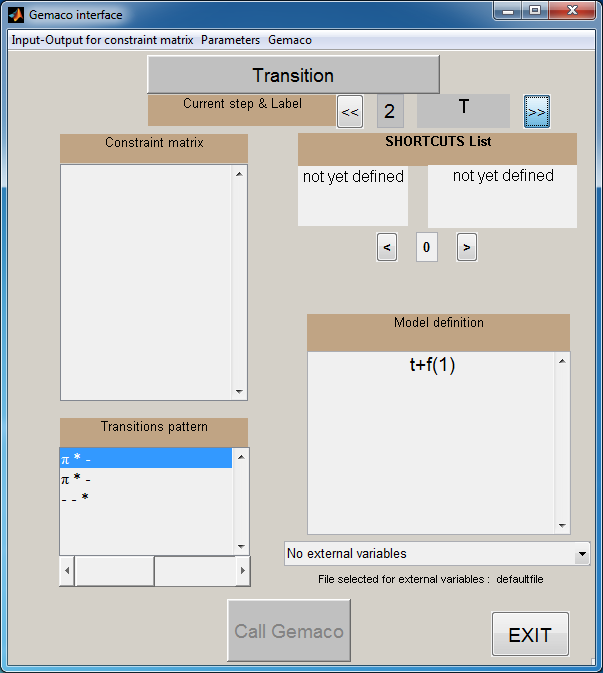


*The event probabilities step is where a new parameter appears: the probability of knowing that an individual is absent from its previous burrow. We assume that this probability is unique over time, etc.: keyword* ‘i’.


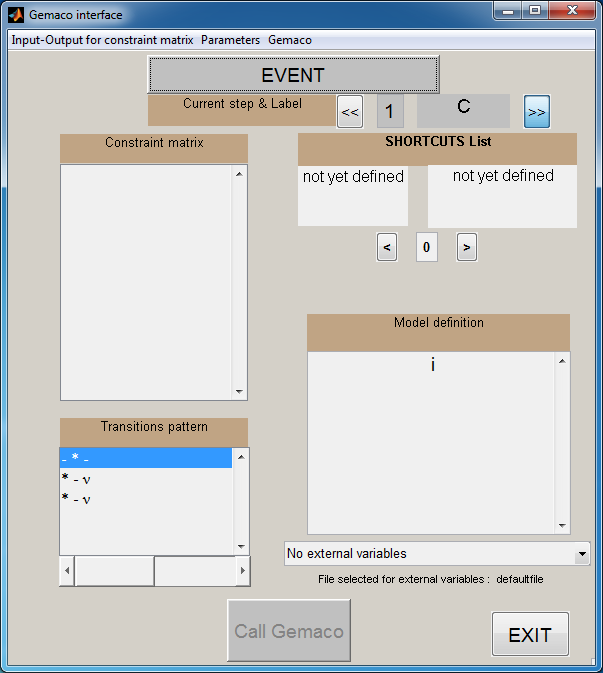


References:

Choquet, R., L. Rouan, and R. Pradel. 2009. Program E–SURGE: a software application for fitting multievent models. Pages 845–865 *in* D. L. Thomson, E. G. Cooch, and M. J. Conroy, editors. Modeling Demographic Processes in Marked Populations. Springer, Berlin, Germany.

Pradel, R., J.E. Hines, J.D. Lebreton, and J.D. Nichols. 1997. Capture-recapture survival models taking account of transients. Biometrics **53**:60–72.

Sanz-Aguilar, A., G. Tavecchia, M. Genovart, J. M. Igual, D. Oro, L. Rouan, and R. Pradel. 2011. Studying the reproductive skipping behavior in long-lived birds by adding nest-inspection to individual-based data. Ecological Applications 21:555–564.
